# The Diagnostic Utility of Induced Sputum Microscopy and Culture in Childhood Pneumonia

**DOI:** 10.1093/cid/cix090

**Published:** 2017-05-29

**Authors:** David R. Murdoch, Susan C. Morpeth, Laura L. Hammitt, Amanda J. Driscoll, Nora L. Watson, Henry C. Baggett, W. Abdullah Brooks, Maria Deloria Knoll, Daniel R. Feikin, Karen L. Kotloff, Orin S. Levine, Shabir A. Madhi, Katherine L. O’Brien, J. Anthony G. Scott, Donald M. Thea, Peter V. Adrian, Dilruba Ahmed, Muntasir Alam, Juliet O. Awori, Andrea N. DeLuca, Melissa M. Higdon, Ruth A. Karron, Geoffrey Kwenda, Eunice M. Machuka, Sirirat Makprasert, Jessica McLellan, David P. Moore, John Mwaba, Salim Mwarumba, Daniel E. Park, Christine Prosperi, Ornuma Sangwichian, Seydou Sissoko, Milagritos D. Tapia, Scott L. Zeger, Stephen R. C. Howie, for the PERCH Study Group, Katherine L. O’Brien, Orin S. Levine, Maria Deloria Knoll, Daniel R. Feikin, Andrea N. DeLuca, Amanda J. Driscoll, Nicholas Fancourt, Wei Fu, Laura L. Hammitt, Melissa M. Higdon, E. Wangeci Kagucia, Ruth A. Karron, Mengying Li, Daniel E. Park, Christine Prosperi, Zhenke Wu, Scott L. Zeger, Nora L. Watson, Jane Crawley, David R. Murdoch, W. Abdullah Brooks, Hubert P. Endtz, Khalequ Zaman, Doli Goswami, Lokman Hossain, Yasmin Jahan, Hasan Ashraf, Stephen R. C. Howie, Bernard E. Ebruke, Martin Antonio, Jessica McLellan, Eunice Machuka, Arifin Shamsul, Syed M.A. Zaman, Grant Mackenzie, J. Anthony G. Scott, Juliet O. Awori, Susan C. Morpeth, Alice Kamau, Sidi Kazungu, Micah Silaba Ominde, Karen L. Kotloff, Milagritos D. Tapia, Samba O. Sow, Mamadou Sylla, Boubou Tamboura, Uma Onwuchekwa, Nana Kourouma, Aliou Toure, Shabir A. Madhi, David P. Moore, Peter V. Adrian, Vicky L. Baillie, Locadiah Kuwanda, Azwifarwi Mudau, Michelle J. Groome, Nasreen Mahomed, Henry C. Baggett, Somsak Thamthitiwat, Susan A. Maloney, Charatdao Bunthi, Julia Rhodes, Pongpun Sawatwong, Pasakorn Akarasewi, Donald M. Thea, Lawrence Mwananyanda, James Chipeta, Phil Seidenberg, James Mwansa, Somwe wa Somwe, Geoffrey Kwenda, Trevor P. Anderson, Joanne Mitchell

**Affiliations:** 1 Department of Pathology, University of Otago, and; 2 Microbiology Unit, Canterbury Health Laboratories, Christchurch, New Zealand;; 3 Kenya Medical Research Institute-Wellcome Trust Research Programme, Kilifi;; 4 Department of Infectious Disease Epidemiology London School of Hygiene & Tropical Medicine, United Kingdom;; 5 Microbiology Laboratory, Middlemore Hospital, Counties Manukau District Health Board, Auckland, New Zealand;; 6 Department of International Health, International Vaccine Access Center, Johns Hopkins Bloomberg School of Public Health, Baltimore, and; 7 The Emmes Corporation, Rockville, Maryland;; 8 Global Disease Detection Center, Thailand Ministry of Public Health–US Centers for Disease Control and Prevention Collaboration, Nonthaburi;; 9 Division of Global Health Protection, Center for Global Health, Centers for Disease Control and Prevention, Atlanta, Georgia;; 10 Department of International Health, Johns Hopkins Bloomberg School of Public Health, Baltimore, Maryland;; 11 International Centre for Diarrhoeal Disease Research, Bangladesh, Dhaka and Matlab;; 12 Division of Viral Diseases, National Center for Immunizations and Respiratory Diseases, Centers for Disease Control and Prevention, Atlanta, Georgia;; 13 Division of Infectious Disease and Tropical Pediatrics, Department of Pediatrics, Center for Vaccine Development, Institute of Global Health, University of Maryland School of Medicine, Baltimore;; 14 Bill & Melinda Gates Foundation, Seattle, Washington;; 15 Medical Research Council: Respiratory and Meningeal Pathogens Research Unit, and; 16 Department of Science and Technology/National Research Foundation: Vaccine Preventable Diseases Unit, University of the Witwatersrand, Johannesburg, South Africa;; 17 Center for Global Health and Development, Boston University School of Public Health, Massachusetts;; 18 Epidemiology, and; 19 International Health, Center for Immunization Research, Johns Hopkins Bloomberg School of Public Health, Baltimore, Maryland;; 20 Department of Biomedical Sciences, School of Health Sciences, University of Zambia, and; 21 Zambia Center for Applied Health Research and Development, Lusaka;; 22 Medical Research Council Unit, Basse, The Gambia;; 23 University of Calgary Cummings School of Medicine, Alberta, Canada;; 24 Department of Paediatrics & Child Health, Chris Hani Baragwanath Academic Hospital and University of the Witwatersrand, Johannesburg, South Africa;; 25 Department of Pathology and Microbiology, University Teaching Hospital, Lusaka, Zambia;; 26 Milken Institute School of Public Health, Department of Epidemiology and Biostatistics, George Washington University;; 27 Centre pour le Développement des Vaccins (CVD-Mali), Bamako;; 28 Department of Biostatistics, Johns Hopkins Bloomberg School of Public Health, Baltimore, Maryland;; 29 Department of Paediatrics, University of Auckland, and; 30 Centre for International Health, University of Otago, Dunedin, New Zealand; 31 Johns Hopkins Bloomberg School of Public Health, Baltimore, Maryland; 32 Bill & Melinda Gates Foundation, Seattle, Washington; 33 Centers for Disease Control and Prevention (CDC), Atlanta, Georgia; 34 The Emmes Corporation, Rockville, Maryland; 35 Nuffield Department of Clinical Medicine, University of Oxford, United Kingdom; 36 University of Otago, Christchurch, New Zealand; 37 ICDDR, b, Dhaka and Matlab, Bangladesh; 38 Medical Research Council, Basse, The Gambia; 39 KEMRI-Wellcome Trust Research Programme, Kilifi, Kenya; 40 Division of Infectious Disease and Tropical Pediatrics, Department of Pediatrics, Center for Vaccine Development, Institute of Global Health, University of Maryland School of Medicine, Baltimore, Maryland and Centre pour le Développement des Vaccins (CVD-Mali), Bamako, Mali; 41 Respiratory and Meningeal Pathogens Research Unit, University of the Witwatersrand, Johannesburg, South Africa; 42 Thailand Ministry of Public Health–CDC Collaboration, Nonthaburi, Thailand; 43 Boston University School of Public Health, Boston, Massachusetts and University Teaching Hospital, Lusaka, Zambia; 44 Canterbury Health Laboratory, Christchurch, New Zealand

**Keywords:** pneumonia, induced sputum, culture, microscopy, children.

## Abstract

**Background.:**

Sputum microscopy and culture are commonly used for diagnosing the cause of pneumonia in adults but are rarely performed in children due to difficulties in obtaining specimens. Induced sputum is occasionally used to investigate lower respiratory infections in children but has not been widely used in pneumonia etiology studies.

**Methods.:**

We evaluated the diagnostic utility of induced sputum microscopy and culture in patients enrolled in the Pneumonia Etiology Research for Child Health (PERCH) study, a large study of community-acquired pneumonia in children aged 1–59 months. Comparisons were made between induced sputum samples from hospitalized children with radiographically confirmed pneumonia and children categorized as nonpneumonia (due to the absence of prespecified clinical and laboratory signs and absence of infiltrate on chest radiograph).

**Results.:**

One induced sputum sample was available for analysis from 3772 (89.1%) of 4232 suspected pneumonia cases enrolled in PERCH. Of these, sputum from 2608 (69.1%) met the quality criterion of <10 squamous epithelial cells per low-power field, and 1162 (44.6%) had radiographic pneumonia. Induced sputum microscopy and culture results were not associated with radiographic pneumonia, regardless of prior antibiotic use, stratification by specific bacteria, or interpretative criteria used.

**Conclusions.:**

The findings of this study do not support the culture of induced sputum specimens as a diagnostic tool for pneumonia in young children as part of routine clinical practice.

Microscopy and culture of sputum specimens are standard techniques commonly used by diagnostic laboratories to evaluate pneumonia in adults. Despite difficulties interpreting results [1], carefully collected and processed sputum specimens have been shown to be useful diagnostic tools in some contexts [2]. Nonetheless, ongoing controversy continues about the value of routinely examining sputum [3–7]. Furthermore, sputum microscopy and culture are not routinely performed in children, who are typically unable to expectorate, making it difficult to obtain specimens [8].

Induced sputum is widely used to investigate lower respiratory infections in immunocompromised adults, especially for diagnosing *Pneumocystis jirovecii* infection [9]. It has also been used to diagnose pneumonia in children from settings with a high prevalence of tuberculosis [10]. However, few studies have collected induced sputum routinely from children with pneumonia. Recent studies of children hospitalized with community-acquired pneumonia from Finland, Kenya, and New Caledonia showed that collection of induced sputum was well tolerated, with a diagnostic yield from culture ranging from 12% to 65% using different interpretative criteria [11–14].

We made a decision to include microscopy and culture of induced sputum as part of the Pneumonia Etiology Research for Child Health (PERCH) study, a large case-control study involving 9 sites in 7 countries from sub-Saharan Africa and south Asia [15, 16]. The expectation was that this approach may help overcome the inherent difficulties with obtaining specimens from the lower respiratory tract in children with pneumonia and facilitate the diagnosis of pneumonia etiology. In an accompanying article we show that low numbers of squamous epithelial cells (SECs) was the best measure of induced sputum quality in children with pneumonia from the PERCH study, as evidenced by low quantities of oropharyngeal flora and higher prevalence of putative pathogens. A large proportion of induced sputum samples met this criterion for good quality, suggesting that most specimens originated from the lower respiratory tract [17]. Here, we evaluate the diagnostic utility of induced sputum microscopy and culture in the PERCH study. A specific objective was to determine whether sputum culture results should be included in the primary etiology analysis of the study. Other companion articles in this supplement focus on the agreement of polymerase chain reaction results between induced sputum and nasopharyngeal/oropharyngeal specimens, safety of induced sputum collection, and utility of induced sputum for diagnosing tuberculosis [18–20].

## METHODS

### Participants

Participants were children aged 1–59 months who were hospitalized with World Health Organization (WHO)–defined severe or very severe pneumonia as part of the PERCH case-control study, details of which have been described elsewhere [21, 22].

### Chest Radiography

Chest radiographs (CXRs) from each child were interpreted by a panel of radiologists and pediatricians trained in the standardized interpretation of pediatric CXRs [23]. CXRs were classified as demonstrating consolidation, other infiltrate, both consolidation and other infiltrate, or as being normal or uninterpretable.

### Specimen Collection

Induced sputum was obtained from cases at enrollment as described in detail in an accompanying article [17].

### Laboratory Methods

Gram stain smears were made from the most visually purulent portion of each induced sputum specimen. The quality of sputum was assessed microscopically as outlined elsewhere [17], and only specimens with <10 SECs per (100×) low-power field (LPF) were included in further analyses. Microorganisms seen in the smear under (1000×) high power were described according to classic Gram stain morphotypes, and relative numbers of each type were recorded within the following categories: 1 (scanty), 2–9 (1+), 10–99 (2+), ≥100 (3+) per representative high-power field.

The most purulent portion of each specimen was inoculated onto sheep or horse blood, chocolate, and MacConkey agars; streaked out using a standard 4-quadrant streaking method; and incubated at 35°C for 48 hours. Cultures were examined at 24 hours and 48 hours, and predominant organisms were identified and quantified according to the furthest quadrant with visible colonies (first quadrant = scanty; second quadrant = 1+; third quadrant = 2+; fourth quadrant = 3+).

In order to standardize reporting across sites, uniform standard operating procedures, on-site training, and internal and external quality checks were established [24].

### Study Definitions The following definitions were used in the study. 

*Sputum Culture Positivity.* Sputum culture results can be interpreted in a variety of ways and no one consistent approach is used by diagnostic laboratories. In general, it is recommended that potential pathogens must be present as a predominant isolate, usually with a compatible Gram stain appearance [25, 26]. For the interpretative criteria used in this study, we reported separately both organisms cultured in any amount and organisms cultured as the predominant isolate with compatible Gram stain morphotype seen on microscopy.

*CXR*+ *Cases and Nonpneumonia Cases.* CXR+ pneumonia was defined by the presence of consolidation and/or other infiltrate on CXR [23]. Given that WHO-defined severe or very severe pneumonia has poor specificity for CXR+ pneumonia, a proportion of the enrolled cases likely did not have true pneumonia. Using baseline clinical and laboratory characteristics and the absence of CXR changes, a group of “control” children unlikely to have pneumonia (nonpneumonia) were identified. Children who met all of the following 3 criteria were defined as “nonpneumonia”: presence of a normal CXR, negative blood cultures, either normal respiratory rate or nonhypoxic in the absence of crackles *or* normal respiratory rate and nonhypoxic in the presence of crackles. We compared pneumonia to nonpneumonia cases for all analyses.

*Prior Antimicrobial Therapy.* Prior antimicrobial therapy was defined as antibiotic activity in serum by bioassay or documentation of antibiotic administration before induced sputum sample collection [27].

*Microbiologically Confirmed Cases.* Cases were defined as having microbiologically confirmed pneumonia if they had a bacterium that was not regarded as a contaminant isolated from blood, lung aspirate, or pleural fluid [27].

### Statistical Analyses

We hypothesized that pneumonia pathogens are more likely to be detected in sputum samples from children with radiographic pneumonia compared to sputum samples from children in the nonpneumonia group. Also, we hypothesized that pathogens that cause pneumonia are likely to be present in high enough quantities that they should be detected by both culture and microscopy, and thus pediatric induced sputum cultures should be interpreted in conjunction with Gram stain findings.

To assess whether pathogen detection was associated with CXR+ pneumonia among hospitalized infants and young children, we calculated the prevalence of specific bacteria cultured from high-quality induced sputum specimens from CXR+ cases and nonpneumonia cases, stratified by site and prior antimicrobial therapy. Comparisons were made using the χ^2^ test. Odds ratios were calculated for associations of pneumonia status with organism presence using different interpretive criteria in order to examine the effect of Gram stain results on observed associations.

### Ethical Considerations

The institutional review board or ethical review committee at each study site institution and at the Johns Hopkins Bloomberg School of Public Health approved the PERCH study protocol. Parents or guardians of participants provided written informed consent.

## RESULTS

Induced sputum culture results were available for analysis from 3772 (89.1%) of 4232 children enrolled in PERCH. Sputum from 2608 (69.1%) met the quality criterion of <10 SECs per LPF; of these, 1162 (44.6%) had radiographic pneumonia, 398 (15.3%) were nonpneumonia cases, and 1048 (40.2%) did not meet either definition.

Of the 1162 CXR+ cases with high-quality induced sputum, 866 (74.5%) had severe pneumonia and 296 (25.5%) had very severe pneumonia. A total of 197 (17.0%) were from the Gambia, 126 (10.8%) from Mali, 233 (20.1%) from Kenya, 194 (16.7%) from Zambia, 198 (17.0%) from South Africa, 142 (12.2%) from Bangladesh, and 72 (6.2%) from Thailand. The median age of these children was 8 months (interquartile range, 4–17), 493 (42.4%) were female, and 90 (7.8%) had human immunodeficiency virus infection. Among this group, 908 (80.6%) had prior antimicrobial therapy before collection of induced sputum, although the frequency varied by site as follows: 43 (21.8%) at the Gambia, 112 (88.9%) at Mali, 213 (91.4%) at Kenya, 190 (97.9%) at Zambia, 178 (89.9%) at South Africa, 104 (73.2%) at Bangladesh, and 68 (94.4%) at Thailand.

Of the 398 nonpneumonia cases, 67 (16.8%) were from the Gambia, 50 (12.6%) from Mali, 157 (39.5%) from Kenya, 48 (12.1%) from Zambia, 22 (5.5%) from South Africa, 28 (7.0%) from Bangladesh, and 26 (6.5%) from Thailand. Relative to the CXR+ cases, the nonpneumonia cases were slightly older (median age 10 months; interquartile range, 3–20), less likely to be female (34.4%), and less likely to have human immunodeficiency virus (1.0%) infection or to have evidence of prior antimicrobial use (68.2%).

Table 1 shows the prevalence of bacteria cultured from induced sputum samples with <10 SECs per LPF from CXR+ cases, by study site, using the 2 culture interpretative criteria (ie, organisms cultured in any amount and organisms cultured as the predominant isolate with compatible Gram stain morphotype). Tables 2 and 3 show the prevalence of organisms cultured from both CXR+ and nonpneumonia cases using the 2 culture interpretative criteria, by antibiotic exposure status. *Haemophilus influenzae*, *Streptococcus pneumoniae*, and *Moraxella catarrhalis* were the predominant organisms isolated when measured as organism detected in any amount (Table 2) or as the predominant organism (Table 3); their prevalence was similar in CXR+ and nonpneumonia cases. *Klebsiella pneumoniae* was more commonly isolated from cases from South Africa and Zambia, although this regional predominance was less evident when the more rigorous interpretative criterion (predominant organisms with compatible Gram stain morphotype) was used (Table 1). There was no predominant organism in 323 (27.8%) high-quality induced sputum specimens from cases with radiographic pneumonia.

**Table 1.** Prevalence of Bacteria Cultured in Any Quantity or as the Predominant Isolate With Compatible Gram Stain From High-Quality Induced Sputum from Children Aged 1–59 Months With World Health Organization–Defined Severe or Very Severe Radiographic Pneumonia, by Study Site

	**Study Site (n, %**)															
	**Kenya** **N** ^**a**^ **= 233**		**Gambia** **N = 197**		**Mali** **N = 126**		**Zambia** **N = 194**		**South Africa** **N = 198**		**Bangladesh** **N = 142**		**Thailand** **N = 72**		**All Sites** **N = 1162**	
**Bacteria**	Any Quantity	Predominant^b^	Any Quantity	Predominant	Any Quantity	Predominant	Any Quantity	Predominant	Any Quantity	Predominant	Any Quantity	Predominant	Any Quantity	Predominant	Any Quantity	Predominant
***Streptococcus pneumoniae***	58 (24.9)	39 (16.7)	126 (64.0)	79 (40.1)	17 (13.5)	10 (7.9)	36 (18.6)	18 (9.3)	47 (23.7)	26 (13.1)	59 (41.5)	49 (34.5)	23 (31.9)	19 (26.4)	366 (31.5)	240 (20.7)
***Staphylococcus aureus***	2 (0.9)	0 (0.0)	6 (3.0)	3 (1.5)	17 (13.5)	4 (3.2)	21 (10.8)	1 (0.5)	32 (16.2)	11 (5.6)	7 (4.9)	3 (2.1)	9 (12.5)	7 (9.7)	94 (8.1)	29 (2.5)
**Other streptococci and enterococci** ^**c**^	0 (0.0)	0 (0.0)	1 (0.5)	0 (0.0)	3 (2.4)	3 (2.4)	0 (0.0)	0 (0.0)	2 (1.0)	1 (0.5)	1 (0.7)	1 (0.7)	0 (0.0)	0 (0.0)	7 (0.6)	5 (0.4)
***Haemophilus influenzae***	86 (36.9)	33 (14.2)	146 (74.1)	105 (53.3)	56 (44.4)	36 (28.6)	62 (32.0)	22 (11.3)	53 (26.8)	10 (5.1)	58 (40.8)	49 (34.5)	12 (16.7)	7 (9.7)	473 (40.7)	262 (22.5)
***Moraxella catarrhalis***	97 (41.6)	70 (30.0)	121 (61.4)	68 (34.5)	30 (23.8)	19 (15.1)	57 (29.4)	29 (14.9)	30 (15.2)	8 (4.0)	6 (4.2)	5 (3.5)	22 (30.6)	16 (22.2)	363 (31.2)	215 (18.5)
**Enterobacteriaceae** ^**d**^																
**All**	11 (4.7)	4 (1.7)	8 (4.1)	3 (1.5)	19 (15.1)	11 (8.7)	24 (12.4)	8 (4.1)	35 (17.7)	6 (3.0)	5 (3.5)	2 (1.4)	1 (1.4)	0 (0.0)	103 (8.9)	34 (2.9)
***Klebsiella pneumoniae* only**	0 (0.0)	0 (0.0)	5 (2.5)	2 (1.0)	7 (5.6)	5 (4.0)	20 (10.3)	6 (3.1)	20 (10.1)	4 (2.0)	2 (1.4)	2 (1.4)	1 (1.4)	0 (0.0)	55 (4.7)	19 (1.6)
**Mixed gram-negative rods**	5 (2.1)	2 (0.9)	10 (5.1)	1 (0.5)	0 (0.0)	0 (0.0)	0 (0.0)	0 (0.0)	6 (3.0)	4 (2.0)	19 (13.4)	8 (5.6)	2 (2.8)	1 (1.4)	42 (3.6)	16 (1.4)
**Other nonfermentative gram-negative rods** ^**e**^																
**All**	3 (1.3)	2 (0.9)	1 (0.5)	1 (0.5)	6 (4.8)	3 (2.4)	2 (1.0)	1 (0.5)	1 (0.5)	0 (0.0)	14 (9.9)	1 (0.7)	0 (0.0)	0 (0.0)	27 (2.3)	8 (0.7)
***Pseudomonas aeruginosa***	2 (0.9)	2 (0.9)	0 (0.0)	0 (0.0)	1 (0.8)	1 (0.8)	0 (0.0)	0 (0.0)	1 (0.5)	0 (0.0)	1 (0.7)	1 (0.7)	0 (0.0)	0 (0.0)	5 (0.4)	4 (0.3)

High-quality induced sputum defined as <10 epithelial cells per low-power field. Radiographic pneumonia defined as any abnormal chest radiograph (consolidation and/or other infiltrates).

^a^Number of cases at each site who had high-quality induced sputum and radiographic pneumonia.

^b^Isolated as the predominant organisms with compatible Gram stain morphotype.

^c^Other streptococci and enterococci includes Streptococci (other than *S. pneumoniae*) and Enterococci species.

^d^Enterobacteriaceae includes *Escherichia coli*, *Enterobacter* species, *Klebsiella* species, *Citrobacter* species, and *Serratia* species, excluding mixed gram-negative rods. *Klebsiella pneumoniae* also reported separately.

^e^Other nonfermentative gram-negative rods includes *Acinetobacter* species and *Pseudomonas* species. *Pseudomonas aeruginosa* also reported separately.

**Table 2.** Prevalence of Bacteria Cultured in Any Quantity From High-Quality Induced Sputum Among Radiographic Pneumonia Cases and Nonpneumonia Cases, by Antibiotic Exposure Status

	**Regardless of Antibiotic Exposure** ^**a**^			**No Antibiotic Exposure**			**Antibiotic Exposure**		
**Bacteria**	**CXR+** ^**b**^ **(N = 1162) n (%)**	Nonpneumonia^**c**^**(N = 398) n (%)**	***P* Value** ^**d**^	**CXR** ^**+**^ **(N = 219) n (%)**	Nonpneumonia **(N = 124) n (%)**	***P* Value**	**CXR** ^**+**^ **(N = 908) n (%)**	Nonpneumonia **(N = 266) n (%)**	***P* Value**
***Streptococcus pneumoniae***	366 (31.5)	149 (37.4)	.03	141 (64.4)	76 (61.3)	.64	210 (23.1)	68 (25.6)	.41
***Staphylococcus aureus***	94 (8.1)	27 (6.8)	.45	13 (5.9)	12 (9.7)	.20	80 (8.8)	15 (5.6)	.10
**Other streptococci and enterococci** ^**e**^	7 (0.6)	3 (0.8)	.72	2 (0.9)	2 (1.6)	.62	4 (0.4)	1 (0.4)	>.99
***Haemophilus influenzae***	473 (40.7)	168 (42.2)	.60	148 (67.6)	84 (67.7)	>.99	302 (33.3)	77 (28.9)	.21
***Moraxella catarrhalis***	363 (31.2)	156 (39.2)	.005	111 (50.7)	55 (44.4)	.26	238 (26.2)	96 (36.1)	.002
**Enterobacteriaceae** ^**f**^									
**All**	103 (8.9)	33 (8.3)	.76	12 (5.5)	5 (4.0)	.62	89 (9.8)	27 (10.2)	.91
***Klebsiella pneumoniae***	55 (4.7)	15 (3.8)	.49	4 (1.8)	3 (2.4)	.71	50 (5.5)	12 (4.5)	.64
**Mixed gram-negative rods**	42 (3.6)	8 (2.0)	.14	12 (5.5)	1 (0.8)	.04	28 (3.1)	7 (2.6)	.84
**Other nonfermentative gram-negative rods** ^**g**^									
**All**	27 (2.3)	11 (2.8)	.58	1 (0.5)	2 (1.6)	.30	26 (2.9)	9 (3.4)	.68
***Pseudomonas aeruginosa***	5 (0.4)	3 (0.8)	.43	0 (0.0)	0 (0.0)	N/A	5 (0.6)	3 (1.1)	.39

High-quality induced sputum defined as <10 epithelial cells per low-power field.

Abbreviation: CXR, chest radiograph.

^**a**^Prior use of antibiotics defined as bioassay positive, antibiotic administration at the referral facility, antibiotic administration prior to induced sputum specimen collection at the study facility.

^b^Radiographic pneumonia (CXR+) defined as any abnormal CXR (consolidation and/or other infiltrates).

^c^Nonpneumonia case defined as a case with a normal CXR, blood culture pathogen negative, and normal respiratory rate *or* nonhypoxic in the absence of crackles, or normal respiratory rate *and* nonhypoxic in the presence of crackles.

^d^ *P* value from χ^2^ or Fisher exact test.

^e^Other streptococci and enterococci includes Streptococci (other than *S. pneumoniae*) and Enterococci species.

^f^Enterobacteriaceae includes *Escherichia coli*, *Enterobacter* species, *Klebsiella* species, *Citrobacter* species, and *Serratia* species, excluding mixed gram-negative rods. *Klebsiella pneumoniae* also reported separately.

^**g**^Other nonfermentative gram-negative rods include *Pseudomonas aeruginosa*, *Acinetobacter* species, *Pseudomonas* species. *Pseudomonas aeruginosa* also reported separately.

**Table 3.** Prevalence of Bacteria Cultured as the Predominant Organism With Compatible Gram Stain From High-Quality Induced Sputum Among Radiographic Pneumonia Cases and Nonpneumonia Cases, by Antibiotic Exposure Status

	**Regardless of Antibiotic Exposure** ^**a**^			**No Antibiotic Exposure**			**Antibiotic Exposure**		
**Bacteria**	**CXR+** ^**b**^ **(N = 1162) n (%)**	Nonpneumonia^**c**^**(N = 398) n (%)**	***P* Value** ^**d**^	**CXR** ^**+**^ **(N = 219) n (%)**	Nonpneumonia **(N = 124) n (%)**	***P* Value**	**CXR** ^**+**^ **(N = 908) n (%)**	Nonpneumonia **(N = 266) n (%)**	***P* Value**
***Streptococcus pneumoniae***	240 (20.7)	94 (23.6)	.23	90 (41.1)	55 (44.4)	.57	139 (15.3)	37 (13.9)	.63
***Staphylococcus aureus***	29 (2.5)	9 (2.3)	>.99	3 (1.4)	4 (3.2)	.26	26 (2.9)	5 (1.9)	.52
**Other streptococci and enterococci** ^**e**^	5 (0.4)	2 (0.5)	>.99	2 (0.9)	1 (0.8)	>.99	3 (0.3)	1 (0.4)	>.99
***Haemophilus influenzae***	262 (22.5)	92 (23.1)	.84	104 (47.5)	60 (48.4)	.91	141 (15.5)	27 (10.2)	.03
***Moraxella catarrhalis***	215 (18.5)	93 (23.4)	.04	62 (28.3)	29 (23.4)	.37	147 (16.2)	61 (22.9)	.01
**Enterobacteriaceae** ^**f**^									
**All**	34 (2.9)	11 (2.8)	>.99	1 (0.5)	1 (0.8)	>.99	32 (3.5)	10 (3.8)	.85
***Klebsiella pneumoniae***	19 (1.6)	6 (1.5)	>.99	1 (0.5)	0 (0.0)	>.99	17 (1.9)	6 (2.3)	.62
**Mixed gram-negative rods**	16 (1.4)	2 (0.5)	.27	2 (0.9)	0 (0.0)	.54	12 (1.3)	2 (0.8)	.75
**Other nonfermentative gram-negative rods** ^**g**^									
**All**	8 (0.7)	7 (1.8)	.07	0 (0.0)	2 (1.6)	.13	8 (0.9)	5 (1.9)	.19
***Pseudomonas aeruginosa***	4 (0.3)	3 (0.8)	.38	0 (0.0)	0 (0.0)	N/A	4 (0.4)	3 (1.1)	.20

High-quality induced sputum defined as <10 epithelial cells per low-power field.

Abbreviation: CXR, chest radiograph.

^**a**^Prior use of antibiotics defined as bioassay positive, antibiotic administration at the referral facility, antibiotic administration prior to induced sputum specimen collection at the study facility.

^b^Radiographic pneumonia (CXR+) defined as any abnormal CXR (consolidation and/or other infiltrates).

^c^Nonpneumonia case defined as a case with a normal CXR, blood culture pathogen negative, and normal respiratory rate *or* nonhypoxic in the absence of crackles, or normal respiratory rate *and* nonhypoxic in the presence of crackles.

^d^ *P* value from χ^2^ or Fisher exact test.

^e^Other streptococci and enterococci includes Streptococci (other than *S. pneumoniae*) and Enterococci species.

^f^Enterobacteriaceae includes *Escherichia coli*, *Enterobacter* species, *Klebsiella* species, *Citrobacter* species, and *Serratia* species, excluding mixed gram-negative rods. *Klebsiella pneumoniae* also reported separately.

^**g**^Other nonfermentative gram-negative rods include *Pseudomonas aeruginosa*, *Acinetobacter* species, *Pseudomonas* species. *Pseudomonas aeruginosa* also reported separately.

Table 4 shows the organisms cultured from high-quality induced sputum samples from the cases with microbiologically confirmed pneumonia. The same organism was isolated from a normally sterile site and induced sputum in a minority of these cases overall; the exception being *M. catarrhalis* for which 4 of 5 cases with positive cultures from a normally sterile site also had this organism isolated from induced sputum.

**Table 4.** Bacteria Cultured From High-Quality Induced Sputum Samples From Children Aged 1–59 Months With Microbiologically Confirmed Pneumonia

**Bacteria Isolated from Normally Sterile Site**		**Same Bacteria Isolated from Induced Sputum**		**Other Bacteria Cultured from Induced Sputum in Any Quantity**									
	**N**	**Isolated in Any Quantity (n, %**)	**Isolated as the Predominant Organism With Compatible Gram Stain (n, %**)	*Haemophilus influenzae*	*Moraxella catarrhalis*	*Streptococcus pneumoniae*	Mixed Gram- Negative Rods	*Pseudomonas aeruginosa*	*Staphylococcus aureus*	*Escherichia coli*	*Enterobacter cloacae*	Streptococcus Group A	*Klebsiella* Species
*Streptococcus pneumoniae*	35	17 (48.6)	9 (25.7)	15	14	N/A	1	2	2	1	0	0	0
*Staphylococcus aureus*	11	4 (36.4)	1 (9.1)	1	3	2	0	0	N/A	0	0	0	0
*Haemophilus influenzae*	12	8 (66.7)	4 (33.3)	N/A	3	5	0	0	0	0	1	1	1
*Moraxella catarrhalis*	5	4 (80.0)	4 (80.0)	2	N/A	2	1	0	0	0	0	0	0
*Klebsiella pneumoniae*	1	0 (0.0)	0 (0.0)	1	1	0	0	0	0	0	0	0	N/A

Microbiologically confirmed pneumonia defined as a bacterium detected by blood culture or polymerase chain reaction/culture in lung aspirates or pleural fluid.

High quality defined as <10 epithelial cells per low-power field.

Table 5 shows the odds ratios associated with CXR+ case status for detection of the major potential pathogen groupings in induced sputum with varying culture interpretative criteria. For all organisms there was either no association or a negative association between CXR+ pneumonia and the isolation of a pathogenic organism from induced sputum. For all organisms there was either no association or a negative association with the isolation of a pathogenic organism from induced sputum and CXR+ pneumonia (Table 5). This pattern was consistent across all levels of interpretative criteria (Supplementary Table 1).

**Table 5.** Odds Ratio Associated With Radiographic Pneumonia Case (N = 1162) Versus Nonpneumonia Case (Reference) (N = 398) as a Predictor of Organism Presence Among Cases With High-Quality Induced Sputum Samples

	***Haemophilus influenzae***		***Streptococcus pneumoniae***		***Staphylococcus aureus***		***Moraxella catarrhalis***		**Other Gram-Negative Rods** ^**a**^	
**Sputum Culture Interpretative Criteria**	**OR (95% CI**)	***P* Value**	**OR (95% CI**)	***P* Value**	**OR (95% CI**)	***P* Value**	**OR (95% CI**)	***P* Value**	**OR (95% CI**)	***P* Value**
Present in any quantity	0.94 (0.75,1.18)	.60	0.77b (0.61,0.97)	.03	1.21 (0.78,1.89)	.40	0.69b (0.54,0.87)	<.01	1.61 (0.91,2.85)	.10
Present in any quantity with compatible Gram stain	0.98 (0.76,1.26)	.87	0.86 (0.67,1.1)	.22	0.97 (0.51,1.85)	.94	0.72b (0.55,0.93)	<.01	1.38 (0.66,2.89)	.39
Present in any quantity without compatible Gram stain	0.91 (0.65,1.27)	.57	0.62b (0.4,0.98)	.04	1.41 (0.78,2.57)	.25	0.77 (0.52,1.13)	.18	1.91 (0.79,4.59)	.15
Predominant organism	0.91 (0.72,1.16)	.46	0.73b (0.57,0.94)	<.01	1.19 (0.72,1.96)	.50	0.67b (0.52,0.86)	<.01	1.42 (0.73,2.77)	.31
Predominant organism with compatible Gram stain	0.97 (0.74,1.27)	.81	0.84b (0.64,1.1)	.21	1.11 (0.52,2.36)	.79	0.72b (0.55,0.95)	.02	1.26 (0.51,3.13)	.62
Predominant organism without compatible Gram stain	0.86 (0.61,1.23)	.42	0.51b (0.31,0.83)	<.01	1.24 (0.65,2.37)	.52	0.68 (0.45,1.04)	.07	1.59 (0.6,4.2)	.35

Abbreviations: CI, confidence interval; OR, odds ratio.

High-quality induced sputum defined as <10 epithelial cells per low-power field. Radiographic pneumonia (CXR+) defined as any abnormal chest radiograph (CXR) (consolidation and/or other infiltrates). Nonpneumonia case defined as a case with a normal CXR, blood culture pathogen negative, and normal respiratory rate *or* nonhypoxic in the absence of crackles, or normal respiratory rate *and* nonhypoxic in the presence of crackles.

^a^Other nonfermentative gram-negative rods include *Pseudomonas aeruginosa*, *Acinetobacter* species, *Pseudomonas* species.

b*P* < .05.

None of the reported findings changed when another, more rigorous, sputum culture interpretative criterion was used, namely, requiring that organisms be isolated in quantities of 2+ or 3+, with or without compatible Gram stain appearance.

## DISCUSSION

The key finding of this study is that culture of a potential pathogen from induced sputum was not clearly associated with radiographic pneumonia in young children compared to a group of hospitalized children unlikely to have pneumonia, even when a sputum quality standard was applied.

When sputum culture is used in pneumonia etiology studies, predefined criteria have been traditionally used to determine results thought to be associated with pneumonia causation [11, 13, 28, 29]. Only high-quality specimens that contain low numbers of SECs are typically accepted for analysis, and potential pathogens are required to be present as a predominant isolate, usually with a compatible Gram stain appearance [25, 26]. These criteria are derived from expert opinion, and their usefulness has been regularly debated [3–7, 30]. Had we applied this traditional approach to using sputum culture results in the PERCH study, the prevalence data shown in the second column of Table 3 would have been simply incorporated into the main etiologic analysis without further scrutiny. However, there was sufficient concern about the reliability of this approach with pediatric induced sputum specimens that we sought additional evidence of diagnostic utility.

Without a suitable comparator gold standard, it is difficult to determine the diagnostic accuracy of sputum culture for identifying pneumonia etiology. In this study, we compared cases of CXR+ pneumonia with a group of patients who (despite fulfilling the WHO case definition for severe or very severe pneumonia) were unlikely to have pneumonia. This approach is based on the assumption that true bacterial pneumonia pathogens will be overrepresented in induced sputa from children with CXR+ pneumonia compared to children unlikely to have pneumonia. We found no clear association between positive sputum culture results and pneumonia status. Indeed, for *S. pneumoniae* and *M. catarrhalis*, microorganisms that can also be part of the normal oropharyngeal flora, isolation tended to be associated with nonpneumonia status, especially in the absence of a compatible Gram stain smear. In addition, among children with microbiologically confirmed pneumonia, culture of induced sputum often detected a different organism than that detected from the sterile-site culture. While it is certainly possible that some of these children had polymicrobial pneumonia, this finding further highlights concerns about the nonspecificity of induced sputum culture in this age group, even when restricted to high-quality specimens that meet a rigorous definition of culture positivity. It may be possible that induced sputum does not adequately capture what is in the lung and/or that there is inevitable contamination with oropharyngeal flora that compromises even high-quality sputum.

It is standard laboratory practice to interpret sputum culture results in conjunction with the Gram stain smear of the same specimen [25, 26]. This follows the principle that, in general, an organism that acts as a pathogen rather than as a colonizer will be present in sufficient quantity to be seen on direct microscopy as well as to be detected by culture. Greater significance is given to organisms that grow in large amounts and are seen in the Gram stain. Despite a trend for the odds ratios of most organisms associated with CXR+ pneumonia to be higher when there was a compatible Gram stain compared to when there was not, this did not alter the fact that no culture results were associated with radiographic pneumonia.

This study has several limitations. Most importantly, the alternative diagnoses in the nonpneumonia cases (all of whom had respiratory symptoms) were largely unknown and there is residual uncertainty about the appropriateness of using this group as a comparator. It is possible that a large proportion of these cases actually did have pneumonia that was yet to be detected radiographically and that the lack of association between sputum culture results and pneumonia may simply indicate misclassification of “nonpneumonia” episodes. Also, nonpneumonia cases were not equally represented across sites [20]. In addition, about three quarters of cases had received antibiotics before collection of induced sputum, and isolation of potential pathogens in specimens from these children was less common than in those who had not received antibiotics. We may not have had the statistical power to detect associations with case status in those who did not receive antibiotics given the relatively smaller number in this group. We were also reliant on an imperfect definition of prior antibiotic use that may have failed to identify all the cases who had received antibiotics [27]. Another potential limitation is interobserver variability in reporting microscopy and culture results. However, uniform standard operating procedures, on-site training, and internal and external quality checks were incorporated into the PERCH study in an effort to standardize reporting across sites, and quality reviews indicated that these procedures were being followed in a consistent manner [24]. Despite these efforts to standardize methods and to collect all relevant data, there is still a subjective element to sputum culture interpretation that may not have been captured by the variables collected and used in the analyses.

In summary, isolation of bacteria from induced sputum did not reflect CXR+ pneumonia in young children hospitalized with WHO-defined severe or very severe radiographic pneumonia. Despite potential confounding by antibiotic use and the limitations of the study design, these findings did not provide sufficient evidence to incorporate induced sputum culture results into the primary etiology analysis of the PERCH study. Furthermore, the findings of this study do not support the culture of induced sputum specimens as a diagnostic tool for pneumonia in young children as part of routine clinical practice.

## Supplementary Material

Supplementary_Materials

## Notes

***Author contributions.*** D. R. M. led the analysis, interpreted results, and drafted the manuscript. N. L. W. performed analyses and assisted with interpretation of results. S. C. M., D. R. F., L. L. H., and S. R. C. H. assisted with interpretation of results and drafting of the manuscript. O. S. L., K. L. O., D. R. F., D. R. M., M. D. K., L. L. H., H. C. B., W. A. B., S. R. C. H., K. K. L., S. A. M., J. A. G. S., D. M. T., S. C. M., and R. A. K. conceived and designed the study and supervised study conduct. P. V. A., J. O. A., A. N. D., M. M. H., G. K., E. M. M., S. M., J. M., D. P. M., J. M., S. M., D. E. P., C. P., O. S., S. S., and M. D. T. were involved in study conduct, data collection, and/or data management. S. L. Z. provided statistical guidance. All authors reviewed and approved the manuscript. D. R. M. had final responsibility for the decision to submit for publication.

***Acknowledgments.*** We offer sincere thanks to the patients and families who participated in this study. We are grateful to the following members of the original study teams for running the studies and collecting data:

***Financial support.*** PERCH was supported by a grant from the Bill & Melinda Gates Foundation to the International Vaccine Access Center, Department of International Health, Johns Hopkins Bloomberg School of Public Health (grant 48968). J. A. G. S. was supported by a clinical fellowship from the Wellcome Trust of Great Britain (098532).

***Supplement sponsorship.*** This article appears as part of the supplement “Pneumonia Etiology Research for Child Health (PERCH): Foundational Basis for the Primary Etiology Results,” sponsored by a grant from the Bill & Melinda Gates Foundation to the PERCH study of Johns Hopkins Bloomberg School of Public Health, Baltimore, Maryland.

***Potential conflicts of interest.*** K. L. O. has received grant funding from GSK and Pfizer and participates on technical advisory boards for Merck, Sanofi Pasteur, PATH, Affinivax, and ClearPath. M. D. K. has received funding for consultancies from Merck, Pfizer, and Novartis and grant funding from Merck. L. L. H. has received grant funding from Pfizer and GlaxoSmithKline. K. L. K. has received grant funding from Merck Sharp & Dohme. S. A. M. has received honorarium for advisory board membership from Bill & Melinda Gates Foundation, Pfizer, Medimmune, and Novartis; institutional grants from GSK, Novartis, Pfizer, Minervax and Bill & Melinda Gates Foundation; and speakers bureau fees from Sanofi Pasteur and GSK. All other authors: No reported conflicts. The authors have submitted the ICMJE Form for Disclosure of Potential Conflicts of Interest. Conflicts that the editors consider relevant to the content of the manuscript have been disclosed.
