# Supplementary material for: The Diagnostic Utility of Induced Sputum Microscopy and Culture in Childhood Pneumonia
Source: Clin Infect Dis. 2017 May 29;64(Suppl 3):S280–8. doi: 10.1093/cid/cix090 (PMC5447842; doi:10.1093/cid/cix090)
Supplement: Supplementary_Materials [file cix090_suppl_Supplementary_Materials.pdf]

Induced Sputum Culture evaluation

Appendix Table 1. Odds ratio associated with CXR+ case (N=1162) versus non-pneumonia case (reference) (N=398) as a predictor of organism presence, stratified by antibiotic use among cases with high quality induced sputum samples

	H. influenzae				*S. pneumoniae*				*M. catarrhalis*				*S. aureus*				OGNR			
	Prior antibiotics -		Prior antibiotics +		Prior antibiotics -		Prior antibiotics +		Prior antibiotics -		Prior antibiotics +		Prior antibiotics -		Prior antibiotics +		Prior antibiotics -		Prior antibiotics +	
Sputum culture interpretative criteria	OR (95% CI)	P value	OR (95% CI)	P value	OR (95% CI)	P value	OR (95% CI)	P value	OR (95% CI)	P value	OR (95% CI)	P value	OR (95% CI)	P value	OR (95% CI)	P value	OR (95% CI)	P value	OR (95% CI)	P value
Present in any quantity	0.99 (0.62,1.59)	.98	1.22 (0.91,1.65)	.19	1.14 (0.72,1.8)	.57	0.88 (0.64,1.2)	.41	1.27 (0.81,1.97)	.30	0.61 (0.45,0.81)	<.01	0.59 (0.26,1.33)	.20	1.62 (0.91,2.86)	.10	3.85 (0.85,17.35)	.08	1.23 (0.66,2.29)	.51
Present in any quantity with compatible Gram stain	0.98 (0.63,1.54)	.94	1.63 (1.1,2.41)	<.01	1.08 (0.69,1.68)	.73	1.15 (0.8,1.65)	.46	1.39 (0.88,2.2)	.16	0.62 (0.45,0.86)	<.01	0.37 (0.1,1.32)	.13	1.39 (0.61,3.19)	.43	1.72 (0.34,8.65)	.51	1.18 (0.51,2.73)	.70
Present in any quantity without compatible Gram stain	1.03 (0.48,2.23)	.94	0.83 (0.57,1.21)	.33	1.23 (0.49,3.1)	.66	0.49 (0.29,0.82)	<.01	0.79 (0.36,1.7)	.54	0.74 (0.47,1.16)	.18	0.84 (0.29,2.43)	.75	1.76 (0.82,3.77)	.15	--	--	1.28 (0.52,3.14)	.59
Predominant organism	0.92 (0.59,1.43)	.71	1.19 (0.86,1.63)	.29	0.85 (0.55,1.32)	.46	0.81 (0.58,1.13)	.22	1.11 (0.69,1.78)	.67	0.59 (0.43,0.79)	<.01	0.42 (0.15,1.16)	.10	1.61 (0.85,3.02)	.14	1.71 (0.18,16.6)	.64	1.18 (0.58,2.39)	.65
Predominant organism with compatible Gram stain	0.96 (0.62,1.5)	.87	1.63 (1.05,2.52)	.03	0.88 (0.56,1.37)	.56	1.12 (0.76,1.65)	.57	1.26 (0.76,2.11)	.37	0.62 (0.44,0.87)	<.01	0.42 (0.09,1.89)	.26	1.54 (0.59,4.05)	.38	1.13 (0.1,12.63)	.92	1.06 (0.39,2.87)	.92
Predominant organism without compatible Gram stain	0.84 (0.36,1.93)	.68	0.83 (0.55,1.24)	.35	0.79 (0.24,2.53)	.69	0.42 (0.25,0.73)	<.01	0.68 (0.27,1.68)	.40	0.65 (0.4,1.04)	.07	0.44 (0.12,1.68)	.23	1.62 (0.71,3.66)	.25	--	--	1.3 (0.49,3.46)	.60

Abbreviations: OGNR, other gram-negative rods; CXR+, chest radiograph positive.
CXR+ defined as radiographic evidence of pneumonia (consolidation and/or other infiltrates).
Non-pneumonia (Non-Pn) case defined as a case with a normal CXR, blood culture pathogen negative, and normal respiratory rate or non-hypoxic in the absence of crackles, or normal respiratory rate and non-hypoxic in the presence of crackles.
Prior use of antibiotics defined as bioassay positive, antibiotic administration at the referral facility, antibiotic administration prior to IS specimen collection at the study facility.
CXR+ N without prior antibiotics = 219 (19.4%); N with prior antibiotics = 908 (80.6%).
Non-pneumonia case N without prior antibiotics = 124 (31.8%); N with prior antibiotics = 266 (68.2%).
Restricted to IS samples with < 10 squamous epithelial cells per low power field.
Other non-fermentative gram-negative rods include *Pseudomonas aeruginosa*, *Acinetobacter* species, *Pseudomonas* species.

Acknowledgements:

PERCH Expert Group. William C. Blackwelder, Harry Campbell, John A. Crump, Adegoke Falade, Menno D. de Jong, Claudio Lanata, Kim Mulholland, Shamim Qazi, Cynthia G. Whitney.

Pneumonia Methods Working Group. Robert E Black, Zulfiqar A Bhutta, Harry Campbell, Thomas Cherian, Derrick W Crook, Menno D de Jong, Scott F Dowell, Stephen M Graham, Keith P Klugman, Claudio F Lanata, Shabir A Madhi, Paul Martin, James P Nataro, Franco M Piazza, Shamim A Qazi, and Heather J Zar.

PERCH Chest Radiograph Reading Panel

Readers: Dr. Kamrun Nahar, Dr. Fariha Bushra Matin, Dr. Claire Oluwalana, Dr. Bernard E. Ebruke, Dr. Joyce Sande, Dr. Micah Silaba Ominde, Dr. Mahamadou Diallo, Dr. Breanna Barger-Kamate, Dr. Nasreen Mahomed, Dr. David P. Moore, Dr. Anchalee Kruatrachue, Dr. Piyarat Suntarattiwong, Dr. Musaku Mwenechanya, Dr. Rasa Izadnegahdar, **Arbitrators:** Dr. Vera Manduku, Dr. John DeCampo, Dr. Marg DeCampo, Dr. Fergus Gleeson.

PERCH Contributors:

Bangladesh: Kamrun Nahar, Arif Uddin Sikdir, Sharifa Yeasmin, Dilruba Ahmed, Muhammad Ziaur Rahman, Muhammad Yunus, Muhammad Al Fazl Khan, Muhammad Jubayer Chisti, Abu Sadat Muhammad Sayeem, Shahriar Bin Elahi, Mustafizur Rahman; **The Gambia:** Michel Dione, Emmanuel Olutunde, Peter Githua, Ogochukwu Ofordile, Rasheed Salaudeen, David Parker; **Kenya:** Shebe Mohamed, Siti Ndaa, Micah Silaba, Neema Muturi, Angela Karani, Sammy Nyongesa, Anne Bett, Daisy Mugo, Salim Mwarumba, Robert Musyimi, Andrew Brent, James Nokes, David Mulewa, Joyce Sande, John Odhiambo, Joshua Wambua, Nuru Kibirige, Caroline Mulunda, Hellen Mjalla, Norbert Katira, Karen Dama, Loice Masha, Christine Mutunga, Mwanajuma Ngama, Stephen Mangi, Riziki Anthony, Mwarua Yubu, Elijah Wakili, Benson Katana, Shoboi Mgunya, Emmanuel Mumba, Benedict Mver, George Kuria, Felix Githinji, Norbert Kihuha, Boniface Jibendi, Tahreni Bwanaali, Agustus Kea; **Mali:** Nana Kourouma, Aliou Toure, Mahamadou Diallo, Breana Barger-Kamate, Mariam Samake, Seydou Sissoko, Abdoul Aziz Maiga, Mariam Samake, Toumani Sidibe, Mariam Sylla, Aziz Diakite, Bassirou Diarra; **South Africa:** Azwidihwi Takalani, Andrea Hugo, Susan Nzenze, Ndulela Titi, Mmabatho Selela, Malebo Motiane, Minah Nkuna, Nonhlanhla Tsholetsane, Sibonsile Moya, Debra Katisi, Tondani Netshishivhe, Lerato Mapetla, Gudani Singo, Simphiwe Gasa, Cece Mgenge, Nozipho Mthunzi, Nombulelo Monedi, Tanja Adams, Shafeeka Mangera, Jeannette Wadula, Peter

Tsaagane, Jenifer L. Vaughan, Sakina Loonat, Martin Hale, Sugeshnee Pather, Mariëtte Middel, Siobhan Trenor, Palesa Morailane, Ntombi Maya, Rene Sterley, Charné Combrinck, Given Malete, Lerato Qoza, Grizelda Liebenberg, Hendrik van Jaarsveld, Zunaid Kraft, Lisa-Marie Mollentze, Lourens Combrinck, Tsholofelo Mosome; **Thailand:** Sununta Henchaichon, Dr. Tussanee Amornintapichet, Dr. Somchai Chuananont, Toni Whistler, Juraiporn Ratanodom, Patranuch Sapchookul, Ornuma Sangwichian, Sirirat Makprasert, Manoon Hirunsalee, Possawat Jorakate, Anek Kaewpan, Duangkamol Siludjai, Apiwat Lapamnouysup, Dr. Wantana Paveenkittiporn, Waraporn Ubonphen, Dr. Peera Areerat, Ms.Yupapan Wannachaiwong, Ms Tewa Faipet, Ms Punnat Natnarakorn, Ms Ahchanan Sacharone, Mr.Winai Makmool, Ms. Kanlaya Sornwong, Ms. Promporn Sansuriwong, Ms. Ratchanida Potiya, Ms. Wasana Hongsawong, Ms.Wipa Matchaikhen, Ms. Thatsanawan Chaiyabil, Ms.Piyapai Wannarach, Ms Chamaiporn Wadeesirisak, Mr. Yuttapong Norapet, Mattana Bangkung, Mr. Barameht Piralam, Sathapana Naorat, Anchalee Jatapai, Prasong Srisaengchai, Dr. Leonard Peruski, Ms.Dawan Phaensoongnoen, Ms.Tussaaorn Klangprapan, Ms.Narawadee Dumrongdee, Ms.Atchara Srithongkham, Mr. Piyawut Noinont, Ms. Pornthip Kamlee, Ms.Siyapa Mongkornsuk; **Zambia:** Justin Mulindwa, Musaku Mwenechanya, John Mwaba, Magdalene Mwale, Julie Duncan, Kazungu Siazele, Muntanga Mapeni, Emily Hammond; **Canterbury Health Laboratory, Christchurch, New Zealand:** Rose Watt, Shalika Jayawardena; **The Emmes Corporation, Rockville, Maryland:** Mark Wolff, Megan Sanza, Omid Neyzari.
